# Comment on: “Night shift work and risk of breast cancer in women: the Generations Study cohort”

**DOI:** 10.1038/s41416-019-0567-6

**Published:** 2019-09-05

**Authors:** Manolis Kogevinas

**Affiliations:** Barcelona Institute for Global Health (ISGlobal), 88 Doctor Aiguader Rd., 08003 Barcelona, Spain

**Keywords:** Risk factors, Breast cancer

Findings on the association of night shift work with breast cancer in the Generations Study were interpreted by the authors as showing no association.^1^ While the authors have done outstanding work to assemble a large cohort that has previously provided valuable information, the analysis on night shift work is probably uninformative. There are several issues regarding exposure misclassification, inappropriate design and statistical analysis that may affect the validity of the interpretation of the findings: (i) The main question requesting whether participants did night shift mixes evening and night work in the same sentence: “Over the last ten years, have you had any jobs that regularly involved work in the late evening or night (between 10 pm and 7 am)”.^1^ Whether participants attributed the bracketed comment to night work or to both evening and night is a matter of interpretation and certainly an ambiguity that should have been avoided. This could result in the “exposed” group including non-exposed workers particularly since evening work is more prevalent than night work. The authors could possibly further disentangle evening from night work, but this is not presented in the methods or online material. In addition, the questionnaire of the study is not openly available and does not allow the reader to evaluate availability of other information; (ii) The study includes mostly mid-age participants who were requested to provide occupational information for the past 10 years prior to enrolment. It is known that there is a decline of prevalence of night shift work by age, hence many participants could have worked as night workers in an earlier period than the 10 years recorded. Participants classified as “unexposed” could, therefore, include an unknown number of persons working night shifts prior to 10 years since enrolment. Point (i) and (ii) would indicate that both the “exposed” and the “unexposed” groups were contaminated, biasing risk estimates towards the null. The extent of this bias is difficult to quantify from the information provided in the paper; (iii) The authors provide risk estimates by duration of night shift work. Because of the left truncation in exposure assessment they could only have included long duration workers if they had survived and entered the 10 year recorded period; this would bias analyses by duration towards the null; (iv) findings in this study that are possibly least biased are those on intensity of exposure that are duration independent; although not necessarily statistically significant, these intensity-based exposure metrics are the most positive in the study and are, surprisingly, discarded by the authors who attribute these findings to chance.

The overall epidemiological evidence on night shift work and breast cancer points to a positive association although this is unlikely to be very strong (in terms of magnitude of the relative risk). Existing epidemiological evidence tends to support intensity-based measures as those most sensitive in identifying a positive dose-response.^2^ In this context, biases such as the ones mentioned above would render the Generations Study uninformative concerning the evaluation of night shift work and breast cancer. All epidemiological studies have biases and the real issue is how important these biases can be. It is difficult to quantify biases without having access to the data and unfortunately the authors did not attempt to quantify them. In fact, they did not even acknowledge them in the text^1^ making the report, as is, uninformative. While large cohorts such as the Generations Study are valuable they also have constraints and, as acknowledged by the authors^1^ this cohort has limited information on the specific exposure examined, i.e. night shift work. In these situations, it is best not to report specific analyses that are probably biased and that further add to a literature on effects of night shift work that is plagued by numerous uninformative studies.

## Data Availability

Not applicable.
